# Magnetoelectric Coupling by Piezoelectric Tensor Design

**DOI:** 10.1038/s41598-019-55139-1

**Published:** 2019-12-16

**Authors:** J. Irwin, S. Lindemann, W. Maeng, J. J. Wang, V. Vaithyanathan, J. M. Hu, L. Q. Chen, D. G. Schlom, C. B. Eom, M. S. Rzchowski

**Affiliations:** 1 Department of Physics, University of Wisconsin-Madison, Madison, Wisconsin 53706 United States; 2 Department of Materials Science and Engineering, University of Wisconsin-Madison, Madison, Wisconsin 53706 United States; 3 Department of Materials Science and Engineering, Pennsylvania State University, University Park, Pennsylvania 16802 United States; 4 Department of Material Science and Engineering, Cornell University, Ithaca, New York 14853 United States; 5 Kavli Institute at Cornell for Nanoscale Science, Ithaca, New York 14853 United States

**Keywords:** Ferroelectrics and multiferroics, Ferromagnetism, Surfaces, interfaces and thin films

## Abstract

Strain-coupled magnetoelectric (ME) phenomena in piezoelectric/ferromagnetic thin-film bilayers are a promising paradigm for sensors and information storage devices, where strain manipulates the magnetization of the ferromagnetic film. In-plane magnetization rotation with an electric field across the film thickness has been challenging due to the large reduction of in-plane piezoelectric strain by substrate clamping, and in two-terminal devices, the requirement of anisotropic in-plane strain. Here we show that these limitations can be overcome by designing the piezoelectric strain tensor using the boundary interaction between biased and unbiased piezoelectric. We fabricated 500 nm thick, (001) oriented [Pb(Mg_1/3_Nb_2/3_)O_3_]_0.7_-[PbTiO_3_]_0.3_ (PMN-PT) unclamped piezoelectric membranes with ferromagnetic Ni overlayers. Guided by analytical and numerical continuum elastic calculations, we designed and fabricated two-terminal devices exhibiting electric field-driven Ni magnetization rotation. We develop a method that can apply designed strain patterns to many other materials systems to control properties such as superconductivity, band topology, conductivity, and optical response.

## Introduction

Magnetoelectric materials systems possess a wide range of applications including non-volatile memories, magnetic field sensors, spintronics, tunable RF circuit elements, tunable optics, and biomedical devices^1–3^. Significant effort has been devoted towards the few known materials exhibiting single-phase room temperature magnetoelectricity, but these materials have drawbacks such as weak magnetoelectric coupling or small electric polarizations^4^. Composite magnetoelectrics, consisting of a ferromagnet coupled to a piezoelectric via strain, are a well-studied alternative to single phase magnetoelectrics. Composite magnetoelectrics have the largest reported magnetoelectric coupling constants and suitable electric polarizations, magnetic coercive fields, and saturation magnetizations. These characteristics make them highly promising inverse magnetoelectric effect device candidates, but up to this point such devices have been challenging to implement in thin-film form^2,5–7^. In this work we design, fabricate, and characterize (001)-oriented, thin-film magnetoelectric membrane heterostructures based on the piezoelectric material [Pb(Mg_1/3_Nb_2/3_)O_3_]_0.7_-[PbTiO_3_]_0.3_ (PMN-PT)^8,9^. Giant piezoelectric coefficients and large electro-mechanical coupling have allowed PMN-PT based composite magnetoelectrics to achieve superior performance, for example in magnetic field sensors^3^. By designing the piezoelectric tensor, we overcome previous limitations intrinsic to thin films, and demonstrate perpendicular electric field control of in-plane magnetization at low bias voltages.

The structure presented here overcomes two critical factors limiting thin-film composite magnetoelectrics. The first limitation arises from substrate clamping that greatly reduces the in-plane piezoelectric response of thin films^10–12^, and the second limitation arises from the in-plane four-fold symmetry of most (001) pseudo-cubic piezoelectrics that precludes the anisotropic in-plane strain necessary for in-plane magnetization rotation. Substrate clamping has limited the majority of research on electric field control of magnetization in composite magnetoelectrics to bulk^2,7^. Nanoscale patterning has been shown to partially address this by relaxing the island through its thickness^13–15^. Membranes are free from substrate clamping^16,17^ and operate at low voltage while still providing for high device density. Special crystalline orientations^18–20^, domain switching^21–23^, and extra top electrodes^24^ have addressed the in-plane symmetry limitation, but our piezoelectric tensor design approach directly transforms biaxial piezoelectric strain into uniaxial strain that reorients in-plane magnetization, eliminating complexity and fabrication challenges. We demonstrate in-plane magnetization reorientation with out-of-plane electric fields, and develop design principles that can be used to generate specific strain patterns.

## Results

### Experimental Approach

**Figure 1:**
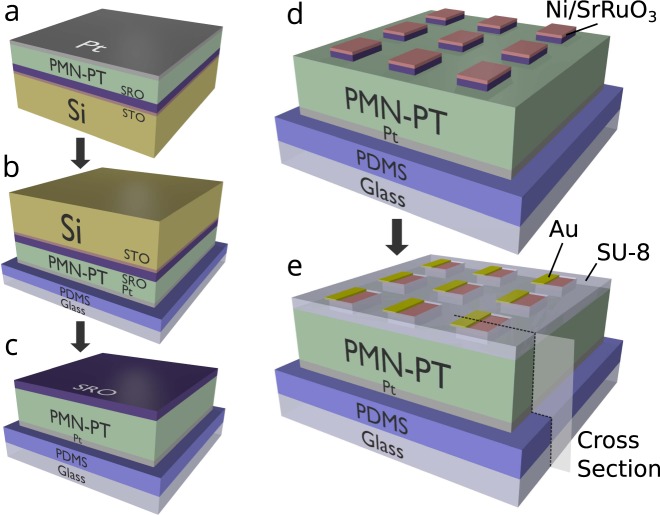
Schematic of the fabrication procedure for membrane magnetoelectric devices. (**a**) PMN-PT/SrRuO_3_/SrTiO_3_/Si thin-film heterostructure with Pt electrode. (**b**) Heterostructure is flipped and attached to PDMS coated glass. (**c**) Si and SrTiO_3_ (STO) are etched off leaving behind a sub-micron membrane. (**d**) Ni is deposited and Ni/SrRuO_3_ (SRO) is patterned into an array of devices. (**e**) A protective coating of SU-8 is applied, and Au contacts are deposited. The cross-section plane is that shown in Fig. 2a.

**Figure 2:**
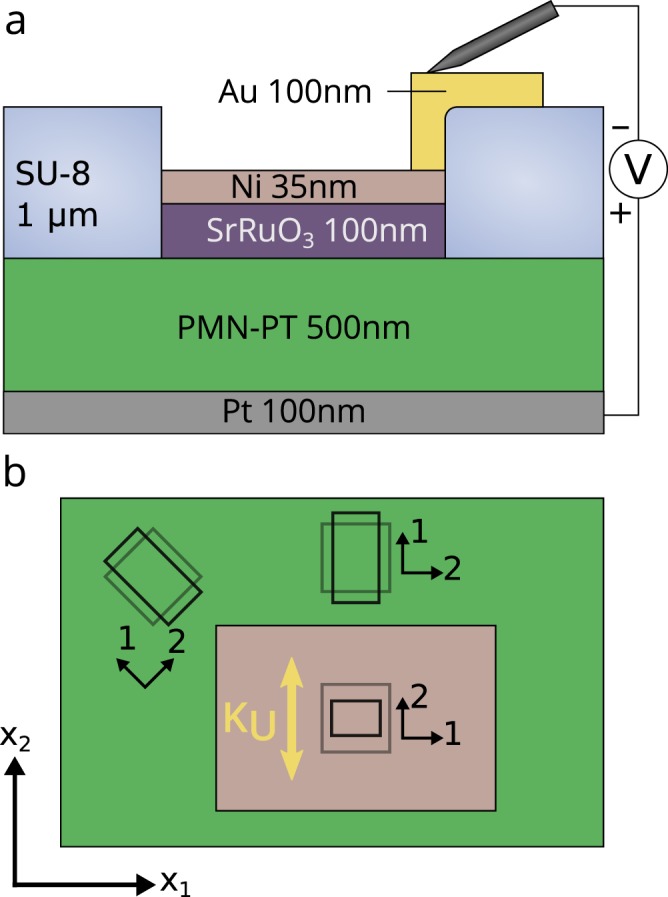
Device schematic. (**a**) Cross section schematic of a completed sample. Positive voltage corresponds to polarization towards the SrRuO_3_. (**b**) Biased regions with aspect ratio A ≠1 generate excess strain along their shorter directions, which induces a magnetic easy direction (gold arrow). The strain distribution in both the biased (pink) and unbiased (green) regions can be considered in terms of local principal strains, shown with small axes indicating directions of principal strains *ε*_1_ and *ε*_2_). The gray squares represent undeformed infinitesimal patches and the black rectangles represent the same patches after deformation due to the piezoelectric response.

The membrane fabrication process starts from an epitaxial PMN-PT / SrRuO_3_ bilayer on SrTiO_3_-buffered Si, and results in a piezoelectric membrane heterostructure on a soft polymer (Polydimethylsiloxane [PDMS]) coated glass slide (Fig. 1, see Materials and Methods for details). Growth of high quality epitaxial PMN-PT/SrRuO_3_/SrTiO_3_ heterostructures on 4° miscut (001)-oriented Si substrates has been previously reported^25^. A continuous Pt film sputtered onto the PMN-PT serves as the bottom electrode. The structure is attached Pt side down to soft PDMS coated glass, then the Si substrate is removed with a XeF_2_ vapor phase etch, and the SiO_2_ by ion-milling. This leaves behind sub-micron thick PMN-PT / SrRuO_3_. The exposed SrRuO_3_ is patterned into top electrodes, defining the PMN-PT biased regions. A 35 nm thick Ni layer is deposited and patterned into regions in which we probe the magnetization rotation via Magneto-optic Kerr Effect (MOKE) measurements. A protective coating of SU-8 polymer and an overlayer of patterned Au allows probe tips to contact individual top electrodes. The cross section of the final heterostructure is shown in Fig. 2a.

Structural, ferroelectric and piezoelectric characterization of the PMN-PT was performed on thin-film and membrane samples. High-resolution X-ray diffraction shows that the biaxiallly strained thin film PMN-PT relaxes towards bulk lattice constants after substrate removal (Supplementary Fig. S1). Release from the substrate also results in a slight increase in the PMN-PT (002) rocking curve from 0.4° to 0.5° due to the lattice constant relaxation (Supplementary Fig. S1). According to polarization-electric field hysteresis loops the PMN-PT has a remanent polarization of 20 μC/cm^2^ and a ferroelectric imprint of 50 kV/cm favoring the polarization pointing towards the SrRuO_3_ (Supplementary Fig. S2).

A key aspect of our membranes is that the PMN-PT layer is continuous, with electrically biased regions (defined by patterned SrRuO_3_ top electrodes) embedded in unbiased PMN-PT. A bias voltage applied between the continuous Pt bottom electrode and the patterned SrRuO_3_ top electrode polarizes only this defined region of the PMN-PT, and we find that the intrinsic isotropic in-plane strain state is transformed by interaction with the surrounding unbiased PMN-PT into the anisotropic strain required to drive in-plane magnetic anisotropy. Anisotropic strain is present both inside and outside of the biased region, and the strain direction is spatially varying (Fig. 2b). We refer to this interaction as boundary clamping and show that it can be used to design an electric field induced strain that controls the in-plane magnetization orientation in the Ni regions. Our measurements of membrane composite magnetoelectrics show electric-field induced uniaxial anisotropy and are in good agreement with our analytical and numerical analyses of the piezoelectric strain tensor in this constrained geometry.

## Experimental Results

Longitudinal magneto-optic Kerr effect (MOKE) magnetic hysteresis loops were used to measure strain-induced magnetic anisotropy in the Ni at different PMN-PT bias voltages. Applying the magnetic field along an easy magnetic direction will result in a square hysteresis loop as the magnetization jumps between orientations parallel and antiparallel to the applied field. Applied field along a hard direction rotates the magnetization away from the easy axis, resulting in a linear hysteresis loop with zero coercivity that saturates at an applied field *H*_sat_. The uniaxial magnetic anisotropy energy density *K*_*U*_ can be estimated from the hard axis data with ${K}_{U}=\frac{{\mu }_{0}}{2}{M}_{{\rm{s}}}{H}_{{\rm{sat}}},$ where *M*_s_ is the Ni saturation magnetization, and assuming coherent rotation^26^.

**Figure 3:**
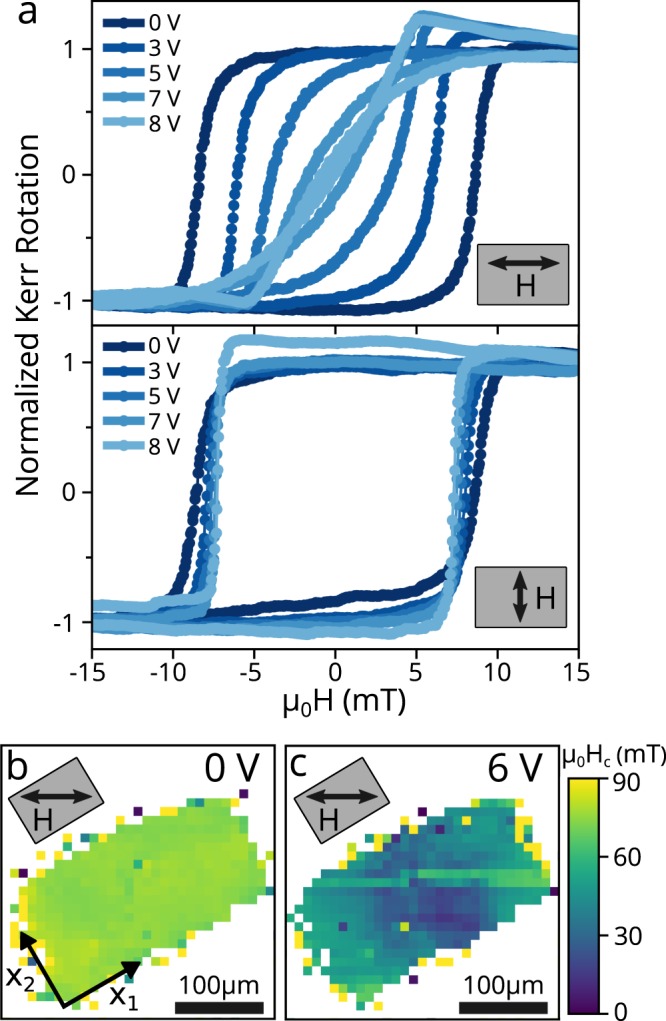
Measurement of Strain-induced Magnetic Anisotropy. (**a**) Magnetic hysteresis loops with the applied magnetic field parallel (top panel) and perpendicular (bottom panel) to the long edge of the pattern. (**b**) Map of coercive field (H_C_) across a Ni island measured with MOKE at zero bias. No magnetic signal was detected at white pixels. (**c**) Coercive field map of the same island with a 6 V applied bias.

Figure 3a shows the bias dependence of MOKE hysteresis loops of a 300 μm by 200 μm Ni/SrRuO_3_ rectangle that serves as top electrode for PMN-PT bias. In the top panel, the applied field magnetic field is along ${\hat{x}}_{1}$, parallel to the long edge of a rectangle. As the applied bias is increased from 0 V to 8 V, the loops close from square to nearly linear, indicating the formation of a magnetic hard direction along ${\hat{x}}_{1}\,$with an anisotropy energy of 1.2 kJ/m^3^. In the bottom panel of Fig. 3a, the measurement field is rotated by 90° to be along ${\hat{x}}_{2}$, parallel to the shorter edge of the pattern. As the applied bias increases, there is a small change in coercive field but no noticeable change in loop shape, showing that the ${\hat{x}}_{2}$ axis remains easy, independent of bias. These two measurements confirm that the piezoelectric strain has induced a new uniaxial anisotropy in the Ni layer along ${\hat{x}}_{2}$. As Ni has a negative magnetostriction constant, ${\hat{x}}_{2}$ must be the most compressively strained direction in the biased region. At zero bias, the hysteresis loops for both field directions (and all others measured but not shown) are identical, indicating no intrinsic anisotropy in this sample. Strain-induced uniaxial magnetic anisotropy is expected to immediately induce a hard-axis response, with a magnetic anisotropy proportional to strain, rather than the incremental closing of the loops observed experimentally. We believe that our experimental results arise from strain-induced anisotropy competing against domain wall pinning, consistent with the relatively large 10 mT Ni coercive field, attributable to growth conditions. Devices on other samples showed an incremental increase in anisotropy energy with increasing bias voltage (Supplementary Fig. S6).

Figure 3c,d show spatial maps of the Ni coercivity measured with MOKE. A complete hysteresis loop was measured with the laser focused at each 10 μm × 10 μm pixel and the magnetic field was aligned 30° from the previously determined strain-induced hard axis direction. Shadowing of the laser by a wire bond prevented measurement with the magnetic field along *x*_1_or *x*_2_ so an intermediate angle was chosen. At zero bias (Fig. 3b), the coercive field is uniform and matches the zero-bias coercive field measured in Fig. 2a. At a 6 V bias (Fig. 3c), the coercive field drops considerably indicating loop closure and a strain-induced magnetic anisotropy. The loops don’t close completely due to the 30° misalignment with the hard axis. The coercivity is lower near the center of the pattern, and higher near the short edges, suggesting a larger anisotropy near the center of the electrode, as expected based on our analysis below.

**Figure 4:**
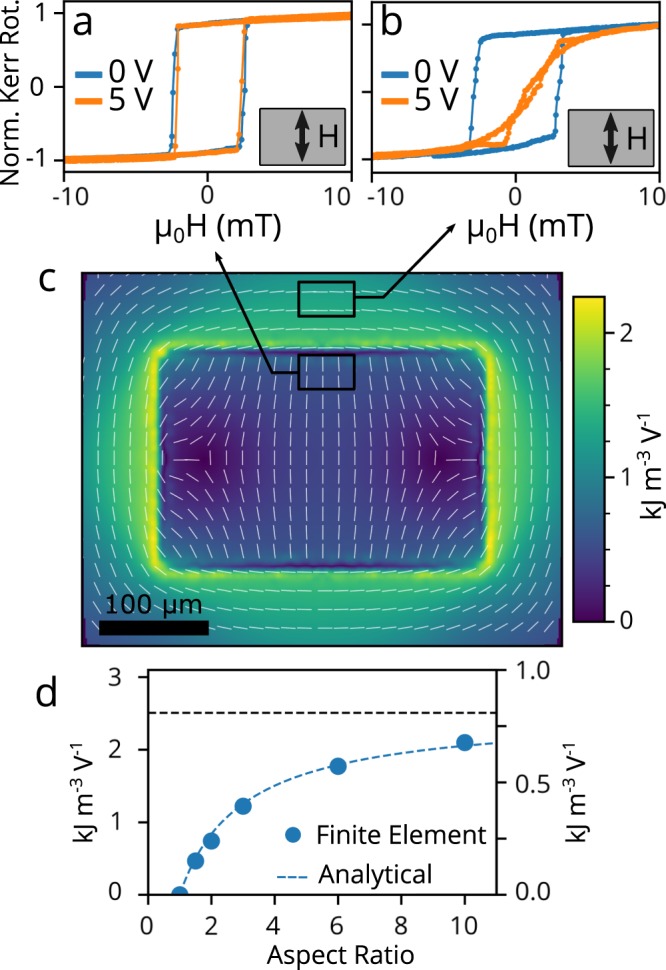
Comparison of Experiment, Simulation and Theory. MOKE hysteresis loops measured on Ni islands placed just inside (**a**) and outside (**b**) a 3:2 biased region at 0 V and 5 V bias and with the magnetic field along ${\hat{x}}_{2}$. (**c**) Induced magnetic anisotropy per applied voltage on and around the biased region. Color represents anisotropy energy and the white lines are the anisotropy axis direction. Black rectangles indicate the experimentally probed regions. (**d**) Aspect ratio dependence of the simulated (circles, left axis) and Eshelby model (dotted lines, right axis) anisotropy energy inside elliptical biased regions. The asymptotic value is shown by the dashed black line.

In addition to the strains within the biased region of the PMN-PT, there is also significant strain outside of the biased region. The strain-induced magnetic anisotropies inside and outside of the biased region are qualitatively different. To probe this difference, we patterned a device with a grid of 60 μm by 80 μm Ni islands, each free to respond independently to local strains, placed on and around a 300 μm by 200 μm SrRuO_3_ electrode. MOKE magnetic hysteresis loops are shown for two nearby Ni islands at 0 V and 5 V, one inside (Fig. 4a) and one outside (Fig. 4b) of the biased region. Both islands have square hysteresis loops at zero bias with the applied field along ${\hat{x}}_{2}$. Due to different growth conditions, the Ni in the two islands in Fig. 4a and b have as-grown magnetic anisotropy along ${\hat{x}}_{2}$, in contrast to the Ni in the sample measured in Fig. 3 which was magnetically isotropic. At 5 V, the Ni island inside the biased region has an unchanged hysteresis loop, matching the behavior of the larger Ni rectangle shown in Fig. 1a (bottom panel). The Ni island outside the bias region, under a 5 V bias, develops an 0.84 kJ/m^3^ anisotropy parallel to the long edge and perpendicular to the anisotropy induced inside the biased region. This 90° difference in anisotropy is consistent with our detailed analysis presented below, but can also be understood qualitatively: when the biased PMN-PT compresses inwards, it stretches the unbiased region. The magnetization in the compressed region aligns parallel to the axis of compression (Fig. 4a, easy axis along ${\hat{x}}_{2}$), and in the stretched region aligns perpendicular to the axis of tension (Fig. 4b, easy axis along ${\hat{x}}_{1}$).

## Discussion

Our magnetoelectric measurements demonstrate that piezoelectric strain is responsible for inducing, via magnetoelasticity^27^, a magnetic easy axis along the shorter direction of rectangular electrodes. This would not occur without the boundary clamping of the biased PMN-PT by the surrounding unbiased PMN-PT. Here we develop an analysis that relates the piezoelectric strain, boundary clamping, and magnetic anisotropy, and which allows for the design of an electric field-dependent magnetic anisotropy pattern in the Ni layer through piezoelectric tensor design.

A bias applied across the thickness of the PMN-PT generates strain in the PMN-PT through the converse piezoelectric effect. Normal (non-shear) strains in cubic piezoelectrics are characterized by two piezoelectric tensor components, *d*_33_ > 0 and *d*_31_ < 0, which in this geometry respectively describe the elongation parallel and perpendicular to the applied electric field. Because of its tetragonal symmetry when polarized along ${\hat{x}}_{3}$, an unconstrained sheet of PMN-PT responds equally along ${\hat{x}}_{1}\,$and ${\hat{x}}_{2}$ (i.e. *d*_31_ = *d*_32_), creating isotropic strain. However, when only a small region of the membrane is biased, its contraction is constrained by the surrounding unbiased PMN-PT, resulting in anisotropic response. We find that the effect of this boundary clamping can be described with effective (subscript *eff*) piezoelectric tensor components of the biased region, with *d*_32,*eff*_ ≠ *d*_31,*eff*_. This modification leads to strain-dependent uniaxial magnetic anisotropy.

This magnetic anisotropy induced by the applied bias depends on the in-plane components of the strain tensor, which are spatially varying due to the boundary clamping. Locally, every two-dimensional strain distribution has a direction of maximum strain and minimum strain, referred to as the first and second principal strain directions. The notation *ε*_1_ and *ε*_2_ is used here to denote the magnitude of the first and second principal strains. In terms of the principal strains, the induced anisotropy energy in the presence of an arbitrary strain distribution is

1 $${K}_{U}=-\,\frac{3}{2}{\lambda }_{S}{Y}_{Ni}({\varepsilon }_{1}-{\varepsilon }_{2})$$

where *λ*_*S*_ and *Y*_*Ni*_ are the saturation magnetostriction constant (−32.9 ppm) and Young’s modulus (220 GPa) of polycrystalline Ni^27^. This means that however complex the strain distribution, it locally induces a uniaxial anisotropy, with direction and magnitude determined by the principal strains of the strain tensor. Here the anisotropy axis is parallel to the second principal strain direction, because this is the most compressed direction and Ni has a negative *λ*_*S*_. Figure 2b schematically shows the principal strains at three infinitesimal regions in the biased and unbiased regions of a piezoelectric membrane. Upon applying a bias, the gray (undeformed) square patches are stretched or compressed into the black rectangular patches, each with its own principal strain directions.

We can estimate the strain difference *ε*_1_ − *ε*_2_ at the center of the biased region from Eq. (1) using the values of *K*_*U*_ from our MOKE hysteresis loops. Considering only strain-induced anisotropies, the hard axis measurement in Fig. 3a gives *ε*_1_ − *ε*_2_ = 11 ppm at 8 V bias, and that of Fig. 4b gives *ε*_1_ − *ε*_2_ = 78 ppm at 5 V bias. The effective piezoelectric constants may also be estimated as the strain difference per applied electric field, giving *d*_31,*eff*_ − *d*_32,*eff*_ = 6.9 pm/V.

### Analysis

We find that strain patterns in our piezoelectric membranes can be understood by building on a continuum elasticity theory first developed by Eshelby^28^ to describe the elastic behavior of precipitates in materials. An exactly ellipsoidal region embedded in an elastic media will strain anisotropically in response to an isotropic internal stress, with the strain exactly uniform inside the ellipsoid. The strain is largest along the shortest axis of the ellipsoid. This is in agreement with our experimental results: the biased regions in our samples undergo uniform stress from their piezoelectric response, and our MOKE measurements indicate that the largest compressive strain lies along the shorter axis of rectangular patterns, in agreement with Eshelby’s model.

Inside an infinite elliptical cylinder with axes *a* and *b*, respectively along ${\hat{x}}_{1}$ and ${\hat{x}}_{2}$, the strain response to an electric field along the cylinder axis is^29^

2 $${\varepsilon }_{ij}={E}_{3}\frac{{e}_{31}}{(a+b){c}_{11}}(\begin{array}{cc}b & 0\\ 0 & a\end{array})={E}_{3}\frac{{e}_{31}}{(1+A){c}_{11}}(\begin{array}{cc}1 & 0\\ 0 & A\end{array})$$

where *ε*_*ij*_ is the strain tensor, *E*_3_ is the electric field, *e*_31_ is the transverse piezoelectric coupling constant (*e*_*ij*_ = *c*_*ik*_ *d*_*kl*_), and the aspect ratio $A=\frac{b}{a}$. The resulting first and second principal strains are *ε*_11_ (along ${\hat{x}}_{1}$) and *ε*_22_ (along ${\hat{x}}_{2}$). The magnetic anisotropy induced by this strain distribution, as a function of aspect ratio and applied electric field, is found from Eqs. (1) and (2) to be

3 $${K}_{U}=-\,\frac{3}{2}{\lambda }_{S}\,{Y}_{Ni}\,{E}_{3}\frac{{e}_{31}}{\,{c}_{11}}\frac{1-A}{1+A}$$

Using bulk materials constants^30^ in this model yields *K*_*U*_ = 1.1 kJ/m^3^ for an 8 V bias across a 3:2 aspect ratio ellipse, close to the measured value 1.2 kJ/m^3^ for our rectangular electrodes. This order of magnitude agreement suggests that far inside the pattern edges the aspect ratio primarily determines the effect of boundary clamping on the electric field induced magnetic anisotropy. According to this analysis, the magnitude of the magnetic anisotropy is independent of the absolute size of the biased region, suggesting that lateral electrode dimensions much smaller than the 100 μm scale used here would still be effective. However, the assumptions of a sharply defined boundary region and a purely out-of-plane electric field break down for lateral electrode sizes smaller than the piezoelectric thickness due to electric field fringing. This would likely modify the effects described here.

Finite element continuum elastic simulations were performed to address the rectangular biased regions used in our experiments, mapping strains and the resulting magnetic anisotropy (Fig. 4c). All layers of the structure shown in Fig. 2a except the Au and SU-8 were included in the simulation, using bulk values for the elastic, piezoelectric and dielectric tensors of PMN-PT^30^. Figure 4c shows the strain-induced magnetic anisotropy energy per applied voltage (color) and anisotropy direction (white lines) on the surface of the PMN-PT layer. The computed anisotropy predominantly perpendicular to the long edge reproduces the experimental results of Fig. 4a,b. The change in direction near the short edge coincides with very small anisotropy magnitude, and so is difficult to detect experimentally. The computed 0.45 kJ m^−3^V^−1^ magnitude in the large central portion of the biased region predicts a 3.6 kJ m^−3^ anisotropy energy density at 8 V, three times the experimental value. The computed anisotropy is largest near the center, consistent with the experimental spatial maps of Fig. 3c.

We also simulated a series of elliptical electrodes with varying aspect ratios to compare with the Eshelby approach. Figure 3d shows that the simulated and analytical anisotropy energies have the same $\frac{A-1}{A+1}$ dependence on aspect ratio. However, the analytic result of Eq. (3) describe an infinite cylinder of PMN-PT, and the simulation the experimental two-dimensional composite sheet. The two *y*-axis scales in Fig. 4d indicate that this difference results in different predicted anisotropy energy magnitudes, but the dependence on aspect ratio is captured by the analytic result.

According to finite element calculations, the area of largest uniaxial strain is just outside of the biased region boundary (Fig. 4c). Analytical solutions^31^ for the strain outside of elliptical precipitate inclusions confirm that the largest uniaxial strains are concentrated on the most curved portion of the boundary. The measured anisotropy direction of the Ni island outside the biased region (Fig. 4b) matches the calculated anisotropy parallel to the long edge (Fig. 4c boxed in black) and the direct phase-field calculation of the magnetization direction (Supplementary Fig. S5) for that location. We did not experimentally find a significant difference in the induced anisotropy energy inside and outside of the biased region, likely due to pre-existing magnetic anisotropy and domain pinning in the Ni that makes *H*_sat_ a coarse method for measuring anisotropy energy. A sharp strain drop off outside the biased region would be desirable in a dense array of electrodes to avoid mechanical “cross-talk”. We estimate that the required separation between devices would be on the order of the electrode’s largest dimension, and less separation is required for higher aspect ratio devices. Far away from the boundary, the exterior strains drop off as 1/|***x***|^2^ (see Supplementary equations S8 to S15).

## Conclusions

The preceding analysis, and that summarized in the Supplementary Information, leads to a set of guidelines for setting magnetoelectric response in piezoelectric membrane composites using piezoelectric tensor design. An elongated single electrode generates, in its interior, uniaxial compressive strain and magnetic anisotropy that increases with aspect ratio, and is predominantly oriented along the short axis. Ellipsoidal biased regions have exactly uniform interior strains (Supplementary Fig. S3), with about sixty percent of the limiting anisotropy value obtained at an aspect ratio of 4:1. Substantial further increases require large increases in aspect ratio. Rectangular regions generate about 20% more uniaxial strain than ellipses of the same aspect ratio (Supplementary Fig. S4), but the strain is less uniform in rectangles. The maximum uniaxial tensile strain is located outside highly curved boundaries and is at least twice as large as the interior uniaxial strain, but at the cost of reduced spatial uniformity (Supplementary equations S8 to S15). In the case of a straight boundary, the exterior magnetic anisotropy is perpendicular to the interior anisotropy. These rules allow for the design of particular anisotropy magnitudes and directions using boundary shape and layout.

Piezoelectric membrane composites are positioned to take advantage of interest in freestanding films and the number of available fabrication techniques^16,17,32,33^. Our results here demonstrate the fundamental principles of piezoelectric tensor design for magnetoelectric coupling in membrane composites, and optimization of the biased region geometry will likely realize even higher ME coupling. As any Si compatible piezoelectric and any ferromagnet that can be deposited at room temperature may be used with our fabrication process, the optimum magnetoelectric response would be obtained with a large piezoelectric response membrane and high magnetostriction magnetic layer^34^. Several theoretical proposals for inducing 180° in-plane magnetization rotation in bulk composite magnetoelectrics using spatially varying electric fields have been put forth^35–37^. Piezoelectric membranes offer an alternative, thin-film platform for realizing such proposals using the design guidelines developed here. We also expect that these structures can be used as sensors, with a piezoelectric readout. Although we have focused on in-plane magnetization manipulation, the biaxial strain present in square or circular devices may also be able to control the out-of-plane magnetization of a ferromagnetic overlayer with perpendicular magnetic anisotropy^38,39^. Additionally, integration of other materials with piezoelectric membranes would allow for electric field control of, for instance, superconducting T_C_^40–42^, band topology^43–45^, conductivity^46^, and optical properties^47^ with designed strain patterns.

## Materials and Methods

### Membrane Fabrication

Figure 1 is the schematic of the fabrication procedure for the PMN-PT membrane devices. Here we will describe the method in detail. Growth of high quality PMN-PT (001) thin films started with a (001) Si wafer with a 4° miscut towards (110) and a 20 nm buffer layer of STO. First, 100 nm of SRO was grown using 90° off-axis rf-magnetron sputtering^48^ at 100 W power and 600 °C. A mixture of Ar and O_2_ gas, flown at 12 sccm and 8 sccm respectively, supplied a working pressure of 200mTorr. PMN-PT films were then grown using a misaligned parallel dual planar magnetron sputtering technique^49^ with substrate rotation with 100 W power at 625 °C. A mixture of Ar and O_2_ gas, flown at 17 sccm and 3 sccm respectively, supplied a working pressure of 500 mTorr for PMN-PT growth. A 100 nm layer of Pt was then deposited on top of PMN-PT by DC magnetron sputtering. The heterostructure was annealed in O_2_ at 300 °C for 30 minutes to reduce residual stress in the Pt film. The Si substrate was then mechanically polished to reduce the thickness from 300 μm down to 100 μm to reduce total etching time during the later XeF_2_ dry etching. After polishing, Polydimethylsiloxane (PDMS), with a 10:1 mixture ratio of monomer to crosslinking agent, was spin-coated onto a glass slide at 5000 rpm for 10 s, resulting in a thickness of approximately 30 um. The thin film heterostructure was then placed Pt-side down onto the uncured PDMS, leaving the Si substrate exposed, and the sample was placed under vacuum for a minimum of 5 hours to remove air bubbles from between the Pt and PDMS layers. After the vacuum treatment, the PDMS was then cured on a hot plate for 1 hour at 100 °C.

Once the PDMS was cured, the sample was ready for Si removal. Prior to XeF_2_ etching of Si, a 15 second plasma etch using CF_4_ and O_2_, flowing at 45 sccm and 5 sccm, respectively, to a pressure of 40mTorr, was performed to remove any moisture on the sample, as well as any native SiO_2_ present on the Si substrate. The Si substrate was then completely removed via XeF_2_ etching. The XeF_2_ etching system was performed in a SPTS Xetch e1 XeF_2_ etcher system. The system exposes the samples to XeF_2_ in a cyclic mode, and the recipe used here was chosen to maximize the etch rate for complete removal of the Si substrate. The recipe exposed the samples to 4 Torr of XeF_2_ for two-minute periods, followed by pumping down to 0.8 Torr between cycles for a continuous etch before the next cycle began. Due to the exothermic nature of the reaction of XeF_2_ with Si, the pressure in the chamber rises during the two-minute etch cycles. When the Si is completely removed, the pressure increase is notably absent during an etch cycle, signaling that the etching is complete.

After Si removal, the STO buffer layer was removed via Ar^+^ ion-milling. The SRO layer was then patterned into various geometries using photolithography and wet etching with a 0.4 M NaIO_4_ solution. A 35 nm layer of Ni was deposited by DC Magnetron sputtering, and photolithography was performed to pattern the Ni with a Transene Ni Etchant Type 1 wet etchant. A SU-8 protection layer was applied by spin-coating at 5000 rpm for 40 s, resulting in a thickness of 2um, followed by photolithography patterning. Finally, 30 nm of Au was deposited via DC Magnetron sputtering and patterned via photolithography, and Transene TFA Au wet etchant was used to make the “lifted” Au electrodes.

### Finite-Element Simulations

Finite element calculations were performed with COMSOL Multiphysics^TM^. Simulations were performed using the layers and thicknesses from Fig. 2a. The sheet of PMN-PT and back electrode were 1.4 μm diameter to simulate a small biased region surrounded by a large unpolarized membrane. No mechanical constraints were applied to any surfaces, simulating an unconstrained membrane. The stiffness tensor and piezoelectric coefficients used for PMN-PT may be found in Table 2 of reference^30^. The stress-charge form of the piezoelectric constitutive relations was used:

$$\nabla \cdot {D}_{i}={\rho }_{f}$$

$$\nabla \cdot {\sigma }_{ij}=0$$

$${D}_{i}={e}_{ikl}{\varepsilon }_{kl}+{\varepsilon }_{0}{\kappa }_{ij}{E}_{j}$$

$${\sigma }_{ij}={c}_{ijkl}{\varepsilon }_{kl}-{e}_{kij}{E}_{k}$$

where *D*_*i*_, *E*_*i*_, σ_*ij*_, *ε*_*kl*_, ρ_*f*_, *e*_*ijk*_, *c*_*ijkl*_, *κ*_*ij*_ are the electric displacement, electric field, stress tensor, strain tensor, free charge density, piezoelectric coupling tensor, stiffness tensor and relative permittivity, respectively. A control simulation was performed with 700 nm of Si added below the Pt back electrode, and with a zero-strain constraint on the bottom face (furthest from the PMN-PT) of Si to simulate substrate clamping. In this simulation, the volume-averaged *ε*_11_ − *ε*_22_ in the Ni film dropped to 0.2 ppm from the unconstrained value of 34 ppm, using a 300 μm by 200 μm rectangular biased region and a 20 kV/cm electric field.

### Longitudinal MOKE Measurements

The sample was mounted between the poles of an electromagnet and a red HeNe (632 nm) laser was reflected off of the sample surface at approximately 45° from normal incidence. The beam was focused to an approximately 10 μm spot using an achromat. The reflected beam’s polarization was rotated to 45° from *p*-polarized with a half-wave plate and then the *s*- and *p*-polarized components were measured with a differential balanced photodetector. The differential signal is proportional to the Kerr polarization rotation. Spatial mapping was achieved by mounting the sample on a two-axis linear piezoelectric motion stage and scanning the sample under the focused beam.

## Supplementary Information

Supplementary Information
