# Supplementary material for: Magnetoelectric Coupling by Piezoelectric Tensor Design
Source: Sci Rep. 2019 Dec 16;9:19158. doi: 10.1038/s41598-019-55139-1 (PMC6914799; doi:10.1038/s41598-019-55139-1)
Supplement: Supplementary file 1 — Supplementary Information [file 41598_2019_55139_MOESM1_ESM.pdf]

Supplementary Information for

Magnetoelectric Coupling by Piezoelectric Tensor Design

J. Irwin[1,†], S. Lindemann[2,†], W. Maeng[2], J. J. Wang[3], V. Vaithyanathan[4], J.M. Hu[2], L.Q. Chen[3], D.G. Schlom[4,5], C.B. Eom[2], M.S. Rzchowski[1,*]

[1]Department of Physics, University of Wisconsin-Madison
Madison, Wisconsin 53706, United States

[2]Department of Materials Science and Engineering, University of Wisconsin-Madison
Madison, Wisconsin 53706, United States

[3]Department of Materials Science and Engineering, Pennsylvania State University, University Park, Pennsylvania 16802, United States

[4]Department of Material Science and Engineering, Cornell University,
Ithaca, New York 14853, United States

[5]Kavli Institute at Cornell for Nanoscale Science,
Ithaca, New York 14853, United States

[†]J.I. and S.L. contributed equally to this work
[*]M.S. Rzchowski
Email: rzchowski@physics.wisc.edu

This PDF file includes:

Figs. S1 to S6
Table S1
Equations S1 to S15

Material Characterization

Structural Characterization of Membrane Devices

The structure of the PMN-PT thin films was measured by a high-resolution four-circle XRD machine (Bruker D8 Discover). Figure 1a shows a θ-2θ scan for a PMN-PT thin film before and after substrate removal. Before substrate removal the heterostructure measured here was: 500 nm PMN-PT /100 nm $SrRuO_3$ / 20 nm $SrTiO_3$/ Si (001). The observed peaks were from the Si, SRO, and PMN-PT layers, but the STO layer was too thin for peaks to be observed. Once the membrane device was completed, the heterostructure measured was: 35 nm Ni / 100 nm SRO / 500 nm PMN-PT / 100 nm Pt / PDMS / glass. The SU-8 and Au were not yet deposited onto this device when it was measured. The SRO and Ni layers are patterned into small features of 40-200 μm with large spaces in between, therefore, the SRO peaks are greatly reduced in intensity and the Si (004) peak vanishes. The major observed peaks only come from the PMN-PT and Pt layers.

After the Si has been etched away, the PMN-PT peaks shift towards lower 2θ values meaning that the out-of-plane lattice parameter has increased due to the lattice mismatch strain relaxation. Figure 1b shows only the region around the (002) peaks, and a reference line has been added to highlight the difference in the PMN-PT peak position once the Si is removed. Table 1 shows the 2θ values and corresponding lattice spacings for the PMN-PT thin film, membrane, and bulk PMN-PT with a composition near the MPB for comparison. Once the membrane has been freed from the Si substrate, the 2θ decreases towards the bulk PMN-PT value, showing that the PMN-PT film grows under tensile in-plane strain due to lattice parameter mismatch with Si substrate and relaxes back towards its bulk value upon substrate removal. An increase in the width of the 2θ peaks accompanies this relaxation.

Figure 1c shows the rocking curves of the PMN-PT 002 peaks before and after substrate removal. For the PMN-PT on the Si substrate, the FWHM = 0.4°. Once the PMN-PT is released for the substrate, the FWHM slightly increases to 0.5°. Figure 1d shows phi scans of PMN-PT 101 peaks for the Film and Membrane devices. The FWHM of the phi scans for the film and membrane are 0.95° and 0.99°, respectively.

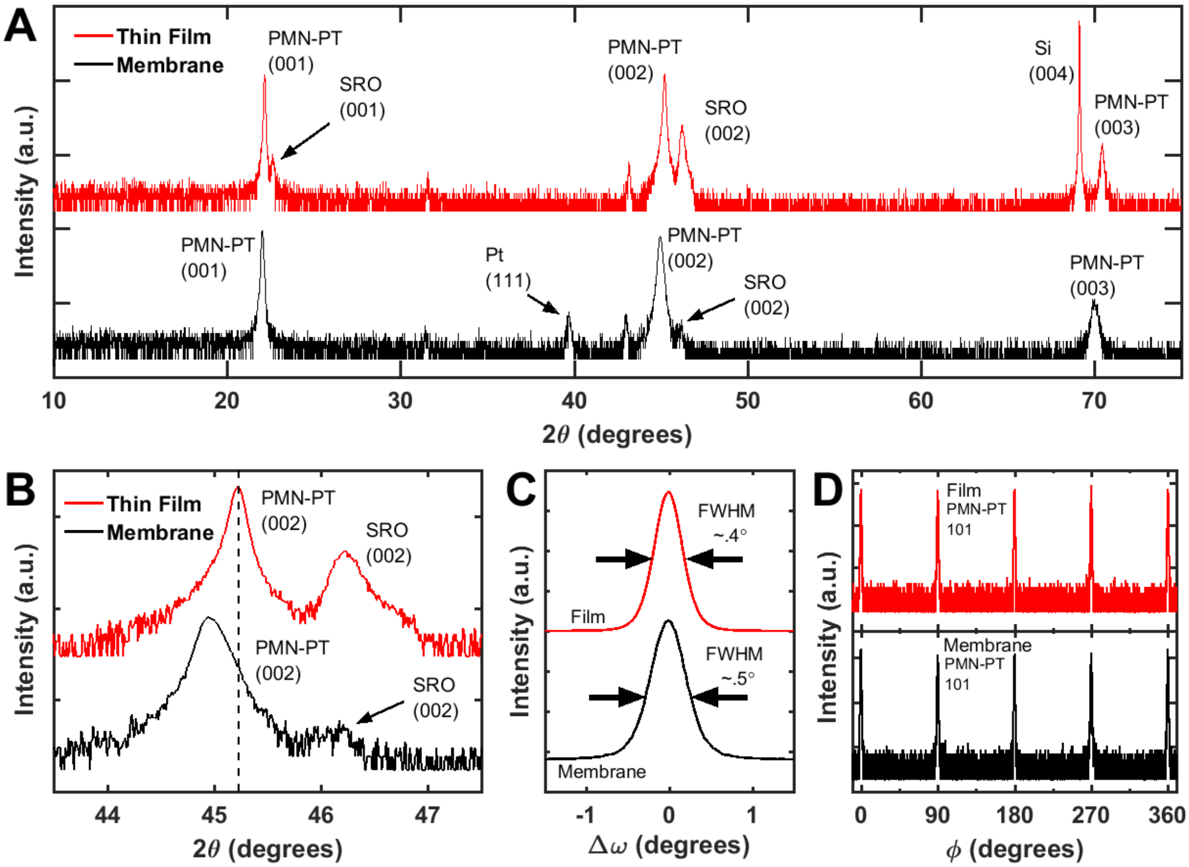

Fig. S1. X-ray Diffraction Characterization of PMN-PT Thin Film and Membrane. (a) Full θ-2θ XRD scan of PMN-PT Thin Film and Membrane. (b) θ-2θ scan region around the (002) PMN-PT peak shifts once the Si substrate is removed. The shift demonstrates the tendency for the PMN-PT to relax towards bulk PMN-PT lattice spacings (Table 1). The membrane device was measured after SRO patterning, therefore the SRO peaks have very little intensity. (c) Rocking Curve of (002) PMN-PT Thin Film Peak and Membrane. There is a slight increase in FWHM upon substrate release. (d) Phi scans of (101) PMN-PT Thin Film and Membrane.

Table S1. Out-of-plane Lattice Spacing of PMN-PT

Sample	2θ	d-spacing
Thin Film	45.225°	4.0068 Å
Membrane	44.960°	4.0292 Å
Bulk*	44.800°	4.0428 Å

*Bulk Value for PMN-PT Compositions near the MPB: (x)PMN-(1-x)PT x ≈ 0.3

Ferroelectric and Dielectric Characterization of PMN-PT Membrane

Figure S2a shows a polarization-electric field hysteresis loops of a PMN-PT membrane released from its substrate, measured at 1 kHz. Figure S2b shows the dielectric constant and loss of the same device, measured with 0.4 kV/cm RMS oscillating electric field at 1kHz. A ferroelectric imprint favoring polarization towards the $SrRuO_3$ (top electrode) is present in all measurements.

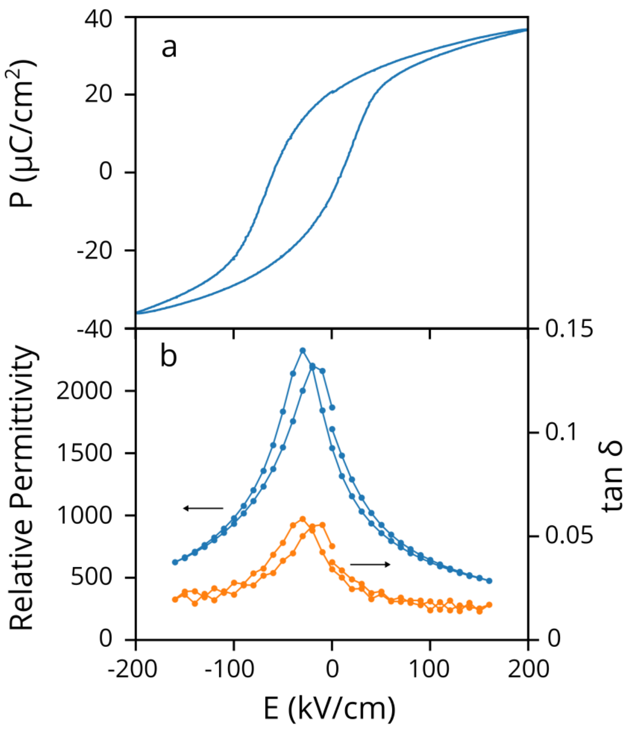

Fig. S2. Ferroelectric and Dielectric Characterization. (a) Polarization-Electric field loop for a membrane device. (b) Permittivity and (c) Loss Tangent measurements of the same membrane device.

Modeling of Piezoelectric Membrane Devices

Anisotropy Map of 2:1 Elliptical Biased Region

Figure S3 shows a finite-element simulation of an elliptical biased region using the same methods and materials parameters as Figure S3 shows that the interior uniaxial strain is exactly uniform, as predicted by the Eshelby model. Outside the biased region the anisotropy direction is approximately tangent to the nearest boundary point. The anisotropy direction does not necessarily rotate by 90° upon crossing from the biased to unbiased region, for example near the ellipse semi-major axis. The rectangular biased region simulation of Figure S3 demonstrated the 90° rotation when crossing both the long and short boundaries, although the anisotropy strength was very low near the short edge, and the direction did not rotate exactly 90° near the corners of the rectangle.

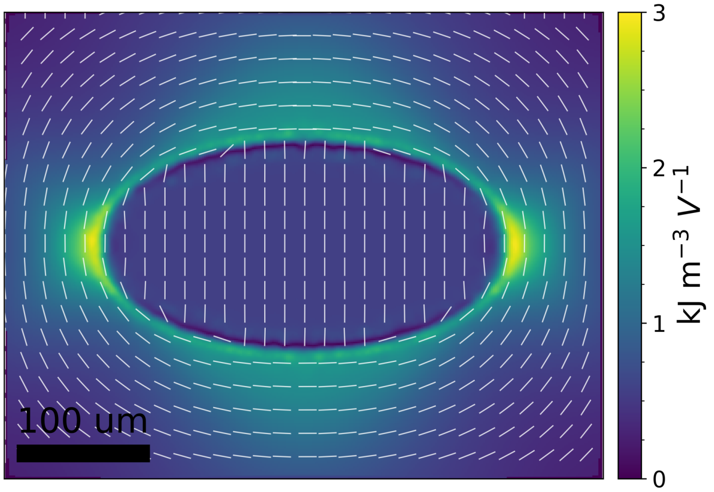

Fig. S3. Finite-element simulation of a 300 μm by 150 μm elliptical biased region.

Strain in Elliptical vs. Rectangular Biased Regions

Finite-element simulation found that the strain in the central region of the biased region is consistently larger in rectangular biased regions than in elliptical ones (Fig. S4). The net effect of the curvature of the elliptical boundaries is to generate uniform interior strain, but at the cost of about a 20% reduction in strain magnitude. Rectangular electrodes have non-uniform strain away from their centers, but the strain per aspect ratio, and per electrode area, is greater.

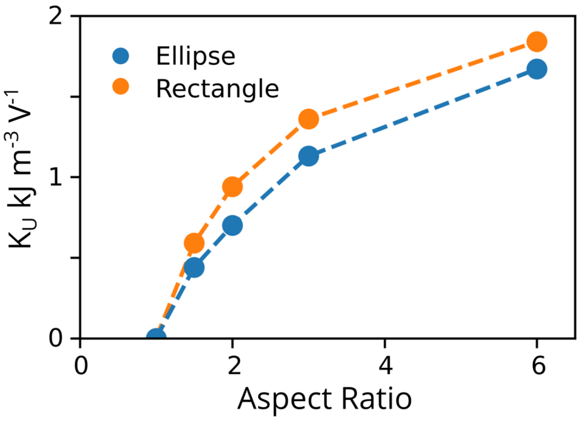

Fig. S4. Comparison of the strain-induced magnetic anisotropy strength per volt at the middle of elliptical and rectangular biased regions, computed with finite-element simulations.

Phase-field Method

In the phase-field model of magnetics, the magnetization is selected as the order parameter to describe the magnetic anisotropy and magnetic domain structures, which can be evolved by the Landau–Lifshitz–Gilbert equation

$$\left(1+\alpha^2\right)\frac{\partial \mathbf{M}}{\partial t} = -\gamma_0 \mathbf{M}\times\mathbf{H}_{\text{eff}} - \frac{\gamma_0\alpha}{M_S}\mathbf{M}\times\left(\mathbf{M}\times\mathbf{H}_{\text{eff}}\right), \tag{S1}$$

where M_S, α, and γ_0 represent the saturated magnetization, damping constant, and gyromagnetic ratio, respectively. The effective field is given by

$$\mathbf{H}_{\text{eff}} = -\frac{1}{\mu_0}\frac{\delta F}{\delta \mathbf{M}} \tag{S2}$$

with F the total free energy including the magnetocrystalline anisotropy energy, exchange energy, magnetostatic energy, external magnetic field energy, and elastic energy

$$F = F_{\text{anis}} + F_{\text{exch}} + F_{\text{ms}} + F_{\text{external}} + F_{\text{elastic}}\,. \tag{S3}$$

In the simulation, F_{anis} is neglected for simplicity due to the isotropic nature of the polycrystalline Ni thin film. The isotropic F_{exch} is determined by the gradient of local magnetization vectors,

$$F_{\text{exch}} = \int_V \zeta\left[\left(\nabla m_1\right)^2 + \left(\nabla m_2\right)^2 + \left(\nabla m_3\right)^2\right] dV\ , \tag{S4}$$

where ζ denotes the exchange stiffness constant.

The magnetostatic energy density F_{ms} can be written as,

$$F_{\text{ms}} = -\int_V \frac{1}{2}\mu_0 M_S\left(\mathbf{H}_d\cdot\mathbf{m}\right)dV. \tag{S5}$$

Here $\mathbf{H}_d$ denotes the stray field, and it can be numerically calculated by employing a finite-size magnetostatic boundary condition previously developed for a 3D array of ferromagnetic cubes[1].

The Zeeman energy of an external magnetic field can be expressed as

$$F_{\text{external}} = -\int_V \mu_0 M_S\left(\mathbf{H}_{\text{ext}}\cdot\mathbf{m}\right)dV\ . \tag{S6}$$

The elastic energy F_{elastic} is written as

$$F_{\text{elastic}} = \frac{1}{2}\int c_{ijkl}\left(\varepsilon_{ij} - \varepsilon_{ij}^{0}\right)\left(\varepsilon_{kl} - \varepsilon_{kl}^{0}\right)dV \tag{S7}$$

through which the magnetoelastic coupling within the Ni island is considered. The total strain ε_{ij} includes a homogeneous part $\overline{\varepsilon_{ij}}$ and an inhomogeneous part $\delta\varepsilon_{ij}$, which can be solved from the mechanical equilibrium equation. The homogeneous strain is assumed to be equal to the in-plane average piezoelectric strain at the surface region of the biased PMN-PT substrate underneath the Ni island.

To solve the phase-field equations of Ni nanoislands, spectral-based approaches are employed with following material parameters for Ni nanoislands: M_s=2.9×10^5 A/m , γ_0=2.2×10^5 m/(A·s), α=0.1, λ_s=−3.3×10^{-5}, c_{11}=247 GP, c_{12}=147 GP, c_{44}=50 GP, ζ =8.2×10^{-12} J/m [1–5]. The discrete grid points of 800Δx×600Δy×Δz with real grid spaces Δx=Δy=5 nm. The real time step Δt of 0.1 ps is used for solving the LLG equation.

Figure S5a shows the magnetization domain structures for Ni islands with zero bias across the PMN-PT membrane, showing multi-domain states of different orientations, indicating that there is no magnetic anisotropy. Figure S5b shows the anisotropic strain distribution computed by the phase-field method, with an 8V bias applied to the 300 μm by 200μm rectangular top electrode. The strain anisotropy transferred to 4 μm by 3 μm Ni islands on top of the membrane depends on their positions relative to the biased region. At position 1, as shown in Fig. S5c, the Ni island magnetization will be switched from multi-domain to single domain with the magnetization parallel to the short edge direction. Similarly, for a Ni island grown at position 2, the simulated magnetization is parallel to the long direction (Fig. S5d), indicating a magnetic easy axis in this direction. The phase-field direct simulation of the magnetoelastic coupling effect on the magnetization agrees with the experimental magnetic easy axis determination from Kerr magnetic hysteresis loops.

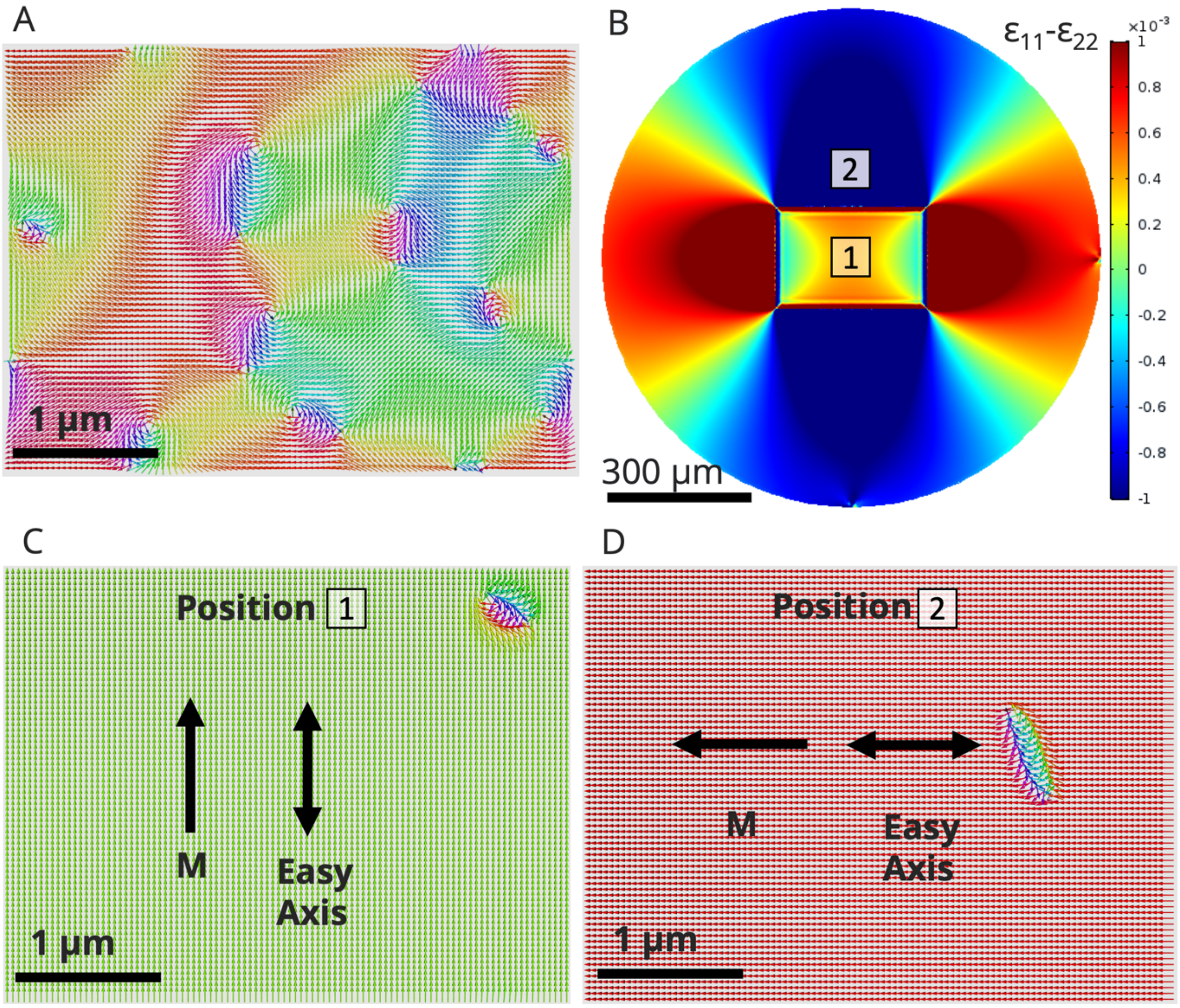

Fig. S5. Micromagnetic simulations of piezoelectric membrane device. (a) Magnetization domain structures for as-grown 4 μm by 3 μm Ni islands on PMN-PT without an applied electric field. (b) Anisotropic strain distribution used for micromagnetic simulations with Ni island positions labelled. Magnetization domain structures for Ni islands located at position 1 (c) and position 2 (d) with 8V bias voltage.

<u>Analysis of Exterior Strain</u>

In this section we will provide analytic support for the design rules relating to magnetic anisotropy inside and outside elliptical biased regions. Jaswon and Bhargava[6] developed an analysis of the elastic response of two-dimensional elliptical elastic inclusions in an elastically isotropic media. Here we use this approach to investigate elliptical biased regions in a piezoelectric membrane, assuming isotropic elastic constants for simplicity. For an elliptical biased region with long axes a along x_1 and short axis b along x_2, confocal elliptical coordinates (ξ, η) with foci at $\pm\sqrt{a^2 - b^2}$ are used to describe the exterior strains. The transformation to Cartesian coordinates is

$$\begin{cases} x_1 = \sqrt{a^2 - b^2} \cosh \xi \cos \eta \\ x_2 = \sqrt{a^2 - b^2} \sinh \xi \sin \eta \end{cases} \tag{S8}$$

Following Jaswon, the exterior strain can be written

$$\varepsilon_{\xi\xi} = -\varepsilon_{\eta\eta} = \gamma \frac{ab}{a^2 - b^2} \frac{e^{2\xi} - \cos 2\eta}{1 + e^{4\xi} - 2e^{2\xi} \cos 2\eta} \tag{S9}$$

$$\gamma = E_3 \, d_{31}(1 + \nu)$$

where ν is the shear modulus of the membrane (the expressions from the reference have been converted into strains using in the limit of plane stress). Along the x_1 axis $\eta = 0$ and along the x_2 axis $\eta = \pi/2$. For these two high symmetry directions the elliptical ξ and η directions align with the Cartesian axes making the induced anisotropy energy

$$K_U = -\frac{3}{2} \lambda_S \, Y_{Ni} |\varepsilon_{\xi\xi} - \varepsilon_{\eta\eta}| = -\frac{3}{2} \lambda_S \, Y_{Ni} |\varepsilon_{xx} - \varepsilon_{yy}| = -3\lambda_S \, Y_{Ni} \, \varepsilon_{\xi\xi}.$$

The boundary of the elliptical biased region is at $\xi = \xi_0$, with ξ_0 determined from any of the following equivalent relations:

$$\cosh 2\xi_0 = \frac{a^2 + b^2}{a^2 - b^2}, \quad \tanh \xi_0 = \frac{b}{a}, \quad e^{2\xi_0} = \frac{a+b}{a-b}. \tag{S10}$$

The maximum and minimum anisotropy energies occur on the $\xi = \xi_0$ contour at $\eta = 0$ and $\eta = \pi/2$, respectively. Combining equations 9 and 10 and converting to anisotropy energy gives

$$K_U|_{\eta=0,\xi=\xi_0} = K_U^{\max} = -3\lambda_S \, Y_{Ni} \, \gamma \frac{A}{A+1} \tag{S11}$$

$$K_U|_{\eta=\pi/2,\xi=\xi_0} = K_U^{\min} = -3\lambda_S \, Y_{Ni} \, \gamma \frac{1}{A+1}, \tag{S12}$$

where the aspect ratio $A = a/b$. In the case of $A = 1$, a circular biased region, everywhere on the boundary $K_U = -3/2 \, \lambda_S \, Y_{Ni} \, \gamma$. For $A > 1$ the maximum and minimum strain respectively are equally above and below the anisotropy value for a circular electrode. The ratio of the maximum to minimum anisotropy on the boundary is equal to the aspect ratio A, and the maximum anisotropy is at the most curved part of the boundary.

The uniform interior magnetic anisotropy can be written in terms of the maximum and minimum anisotropy energies on the boundary. Again, following Jaswon's solution, the anisotropy energy inside the biased region can be written

$$K_U^{int} = -\frac{3}{2} \lambda_{sat} E_{Ni} \left(e_x^{int} - e_y^{int} \right) = -\frac{3}{2} \lambda_{sat} E_{Ni} \, \gamma \frac{A-1}{A+1}, \tag{S13}$$

$$K_U^{int} = \frac{1}{2} \left(K_U^{\max} - K_U^{\min} \right). \tag{S14}$$

In the limit of large A, $K_U^{\min}$ goes to zero and the interior strain-induced anisotropy is half of that just outside of the most highly curved boundary. The ratio of peak boundary anisotropy to interior anisotropy is therefore always greater than or equal to two.

For applications involving a dense array of electrodes, it is desirable for the strain from a single device to be isolated from that of neighboring devices. Far from the boundary the magnitude of the strain decreases as $1/r^2$. To see this, let $\eta = \pi/4$ in equations (S8) and (S9) and simplify to

$$\varepsilon_{\xi\xi} = \frac{\gamma}{2}\frac{ab}{a^2-b^2}\frac{1}{\cosh 2\xi} = \frac{\gamma}{2}\frac{ab}{a^2-b^2}\frac{1}{\cosh^2\xi+\sinh^2\xi} = \frac{\gamma}{4}\frac{ab}{r^2}. \quad \text{(S15)}$$

Although this was derived for a particular value of η it holds for arbitrary η, as contours of constant ξ are circular at large ξ, and strains are constant along the boundary of circular biased regions. Nearer to the boundary, equations (S8) and (S9) can be directly used to compute the strain fields as a function of distance from the boundary. At an aspect ratio of 2:1, the strain drops off to 10% of its peak value within a distance *2a* from its boundary. Strain in higher aspect ratio electrodes with a fixed semi-major axis drops off even faster, as a smaller total volume of material is being polarized under the electrode. At a 6:1 aspect ratio, the strain drops to 10% of peak at around *a/2*. Thus, higher aspect ratio devices provide twin benefits of higher strain and faster strain drop off for less strain cross-talk.

Supplementary Magnetoelectric Characterization

<u>Voltage Control of Magnetic Anisotropy Energy</u>

After an easy axis has formed along the short direction of a rectangular device, excess strain produced by further increases in the bias voltage increases the magnetic anisotropy energy. This can be measured by extracting K_U from hard axis (applied magnetic field along $\hat{x}_1$) MOKE magnetic hysteresis loops at a series of bias voltages. We find a linear increase in anisotropy energy with slope $dK_U/dV = 0.3$ kJm^{-3}V^{-1}. This value is comparable to the 0.45 kJm^{-3}V^{-1} predicted by the finite element model. It was not possible to estimate K_U in this device below 2 V, as the MOKE hysteresis loops were not fully formed hard axis loops, due to the presence of magnetic anisotropy in the as-grown Ni.

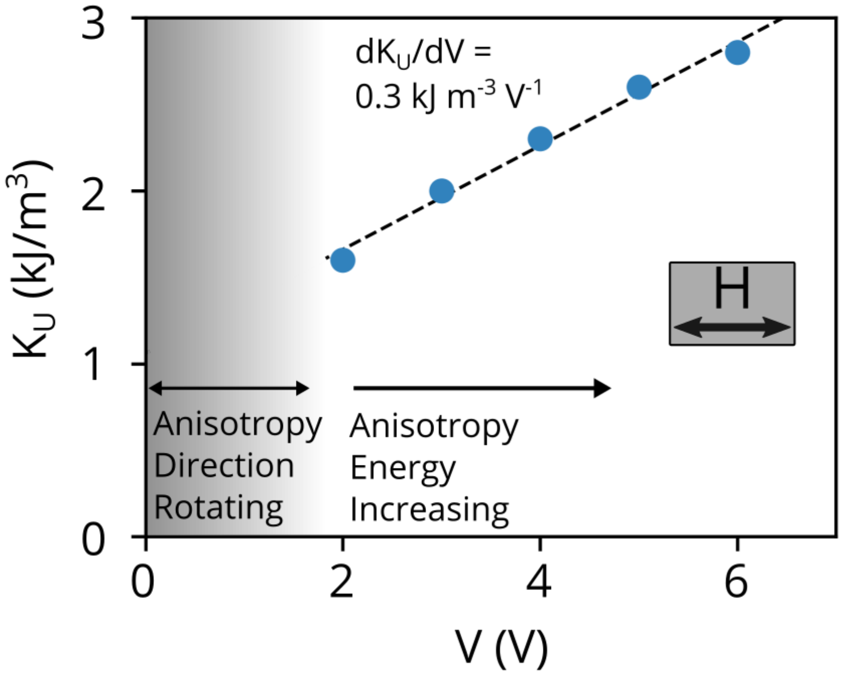

Fig. S6. Anisotropy energy versus bias voltage for a 300 μm by 200 μm biased region covered in continuous Ni, measured via MOKE. K_U was estimated from H_{Sat} of hard axis hysteresis loops. The shaded region indicates bias voltages which did not produce hard axis magnetic hysteresis loops. Inset drawing shows applied field direction relative to device orientation.

References

1. Schabes, M. E. & Aharoni, A. Magnetostatic Interaction Fields for a Three-Dimensional Array of Ferromagnetic Cubes. *IEEE Trans. Magn.* 23, 3882–3886 (1987).
2. Ghidini, M. *et al.* Non-volatile electrically-driven repeatable magnetization reversal with no applied magnetic field. *Nat. Commun.* 4, 1453 (2013).
3. Walowski, J. *et al.* Intrinsic and non-local Gilbert damping in polycrystalline nickel studied by Ti : Sapphire laser fs spectroscopy. *J. Phys. D. Appl. Phys.* 41, 164016 (2008).
4. Wang, J. J. *et al.* Full 180° magnetization reversal with electric fields. *Sci. Rep.* 4, 7507 (2014).
5. Michels, A., Weissmüller, J., Wiedenmann, A. & Barker, J. G. Exchange-stiffness constant in cold-worked and nanocrystalline Ni measured by elastic small-angle neutron scattering. *J. Appl. Phys.* 87, 5953–5955 (2000).
6. Jaswon, M. A. & Bhargava, R. D. Two-dimensional elastic inclusion problems. *Math. Proc. Cambridge Philos. Soc.* 57, 669–680 (1961).
